# Which Stage of Mouse Embryos Is More Appropriate for Vitrification?

**DOI:** 10.22074/ijfs.2016.5086

**Published:** 2016-11-01

**Authors:** Nasibeh Ghandy, Abbas Ali Karimpur Malekshah

**Affiliations:** 1 Department of Anatomy, Faculty of Medicine, Mazandaran University of Medical Sciences, Sari, Iran; 2 Molecular and Cell Biology Research Center, Department of Anatomy, Faculty of Medicine, Mazandaran University of Medical Sciences, Sari, Iran

**Keywords:** Vitrification, Embryo, Preimplantation

## Abstract

**Background:**

Vitrification has been shown as one of the most effective methods of cryopreservation for mammalian embryos. However, there is no consensus which stage of embryonic development is the most appropriate for vitrification with subsequent maximal development after thawing. This study was carried out to explore and compare the effect(s) of vitrification on mouse 2-cell, 4-cell, 8-cell, morula and blastocyst stage embryos and subsequent blast formation and hatching after thawing.

**Materials and Methods:**

In this experimental study, 2-cell embryos were obtained from the oviducts of super ovulated female NMRI mice. Some embryos were randomly selected and vitrified through a two-step media protocol and cryotop. Other embryos were cultured to assess their development. During the ensuing days, some of these cultured embryos were vitrified at 4-cell, 8-cell, morula and blastocyst stages. After 10 to 14 days, the embryos were thawed to assess their survival and also cultured to determine the rate of blastocyst formation and hatching. The results were analyzed using one-way ANOVA and Tukey’s post-hoc tests.

**Results:**

There was no significant difference in the survival rates of vitrified embryos at 2-cell, 4-cell, 8-cell, morula and blastocyst stages after thawing (P>0.05). The blastocyst formation rate of vitrified 8-cell embryos was significantly higher than that of 2-cell embryos (P<0.05). The hatching rate of vitrified 4-cell, 8-cell and blastocysts were significantly higher than that of 2-cell embryos (P<0.05).

**Conclusion:**

Vitrification is suitable for cryopreservation of all stages of mouse embryonic development. However, the best tolerance for vitrification was observed at 4and 8-cell stages of development. Accordingly, the development of vitrified embryos to blastocysts, following thawing, was most efficacious for 4 and 8-cell embryos. Compared to mouse 2-cell embryos, embryos vitrified as blastocysts had the highest rate of hatching.

## Introduction

Cryopreservation of embryo and oocyte are two valuable methods, used to increase the success of infertility treatment. There are two common methods, used for the cryopreservation of embryos: slow freezing and vitrification (1). In slow freezing, embryos are exposed to a gradual decrease in temperature, and are then transferred to liquid nitrogen for storage. 

Vitrification was initially reported in 1985 (2). In this method, the concentration of cryoprotectants is increased and embryos are directly plunged into liquid nitrogen so that they are cooled at a very high speed rate (over 20,000°C/ minute). In this situation, intraand extra-cellular liquids become solid without ice crystal formation. This kind of freezing, as a result, is called vitrification. 

High speed cooling and warming rates are the most critical factors to preserve embryos during the process, which can prevent the formation of ice crystals in the intraand extra-cellular space (3). This method has gained growing worldwide recognition among experts in assisted reproductive technology (ART) laboratories since it was initially used by researchers in an attempt to demonstrate the practicality of vitrification technique, which is commonly used for the cryopreservation of mammalian embryos. This can be attributed to the fact that vitrification has several noticeable merits, which can make it distinct from the conventional slow-freezing method (4). Notable advantages of vitrification over the conventional slow-freezing method are that is simpler, less expensive, more efficient and rapid. This can lead to higher survival and developmental rates when compared to the results of slow-freezing method (5,6). 

In one study has demonstrated that the efficiency of embryo cryopreservation depended not only on the cryopreservation method, but also on the developmental stage of the embryos (7). 

Although several previous studies have already attempted to systematically evaluate the most suitable developmental stage for mouse embryo cryopreservation, there are conflicting results in the literature. Some studies have concluded 2-cell stage (8) is the best for vitrification while the others have noted 8-cell (4), morula (9) or blastocyst (1) as the most optimal stage for embryo cryopreservation and cryotolerance. Moreover, there have been a limited number of published studies investigating the reaction of different stages of mammalian embryo to the stress of cryopreservation and thawing (1,4,8,16). 

With the limited number of published studies and the conflicting reports in mind, we decided to carry out our investigation. In this study, we vitrified mouse embryos at various developmental stages (2-cell, 4-cell, 8-cell, morula and blastocyst) through the cryotop method; so that we could determine the optimal embryonic developmental stage for cryopreservation. It is recognized that the results obtained through mouse embryos could not be extrapolated to human embryos. However, it is hoped that the findings of the current study could provide some empirical insights for prospective researchers with regard to the choice of an optimal developmental stage for the vitrification of human embryos. 

## Materials and Methods

### Experimental design

In this experimental study, 2-cell stage mouse embryos were obtained from 6 to 8 weeks-old NMRI female mice superovulated with 7/5 IU of pregnant mare serum gonadotropin (PMSG, Hipra, Spain) given intraperitoneally. The process was followed by intraperitoneal injection of 7/5 IU of human chorionic gonadotropin (hCG, LG life sciences, Korea) after 48 hours. These mice were then mated with adult male mice of the same strain immediately after the injection of hCG, and were checked for mating the following day morning. The mated female mice were sacrificed by cervical dislocation 44-48 hours after the hCG injection, their oviducts were removed, and the 2-cell embryos were flushed from oviducts. This procedure was performed at room temperature (25°C) and the flushing medium was comprised of a Ham’s F10 with HEPES (Ham’s F10-HEPES, Sigma, Germany) and 20% Human Serum Albumin (HSA, Bio test, Germany). It is worth mentioning that only morphologically normal embryos were used for the sake of this study. 

After the initial wash in flushing medium, 2-cell embryos were randomly divided into two groups. The embryos in the first group were vitrified, and those of the second group were transferred to T6 medium containing 10% HSA for continuous culture at 37°C, in the air with 6% CO^2^humidified incubator. The embryo culture process lasted for four days, following 52-54, 66-68, 72, 96 hours after hCG injection, embryos at 4-cell, 8-cell, Morula, Blastocyst stage, respectively, were isolated from culture medium, and they were subsequently vitrified. 

### Vitrification

Mouse embryos at 2-cell, 4-cell, 8-cell, morula and blastocyst stages were vitrified by a twosteps procedure through the cryotop as a carrier, as described by Kuwayama et al. (5). Embryos were initially equilibrated in vitrification solution 1 (VS1) comprising 7.5% (v/v) ethylene glycol (EG, Sigma, Germany) and 7.5% (v/v) dimethyl sulfoxide (DMSO, Sigma, Germany) at room temperature for 8 to 10 minutes. They were subsequently placed in vitrification solution 2 (VS2) consisting of 15% (v/v) EG, 15% (v/v) DMSO and 0.5 mol/L sucrose (Sigma, Germany). In less than 2 minutes, 4-5 embryos in minimal vitrification solution were placed on the inner surface of the cryotop carrier (Kitazato, Japan). After that, the cryotop was vertically dipped into liquid nitrogen. Then it was inserted into a protective straw-cap before it was cryostored in liquid nitrogen. 

### Thawing

After cryo-storage for 10-14 days, the embryos were thawed. Briefly, the cryotop containing the embryos were removed from the protective straw-cap and dipped into thawing solution 1 (TS1), containing 1.0 mol/L sucrose, at 37°C. After 1 minute equilibration in TS1, the embryos were moved into thawing solution 2 (TS2), containing 0.5 M sucrose, for 3 minutes. Subsequently, embryos were washed for 5 minutes in three drops of Ham’s F10-HEPES medium with 20% HSA at 37°C (4). After that, embryos were cultured in T6 medium with 10% HSA under mineral oil at 37°C, in air with 6% CO_2_humidified incubator. 

The survival rate of embryos was assessed by observing the lightness and intactness of blastomers and zona pellucida. Development of the embryos were maintained and monitored until they reached the blastocyst and hatching stages. The survival rate, blastocyst formation, and the hatching rate were all recorded and compared among the groups. 

### Statistical analysis

The data were statistically analyzed using the Statistical Package for Social Sciences (SPSS, Version 15.0). One-way ANOVA and Tukey’s post-hoc tests were used to compare and analyze the post-vitrification survival rates, blastocyst formation rates, and hatching rates among the experimental groups. Results have been reported as mean ± SD. A P value<0.05 was considered significant. 

### Ethical considerations

This project has been approved by the Ethic Committee of Mazandaran University of Medical Sciences (Sari, Iran) with code No. 91-108. 

### Results

**Fig.1:**
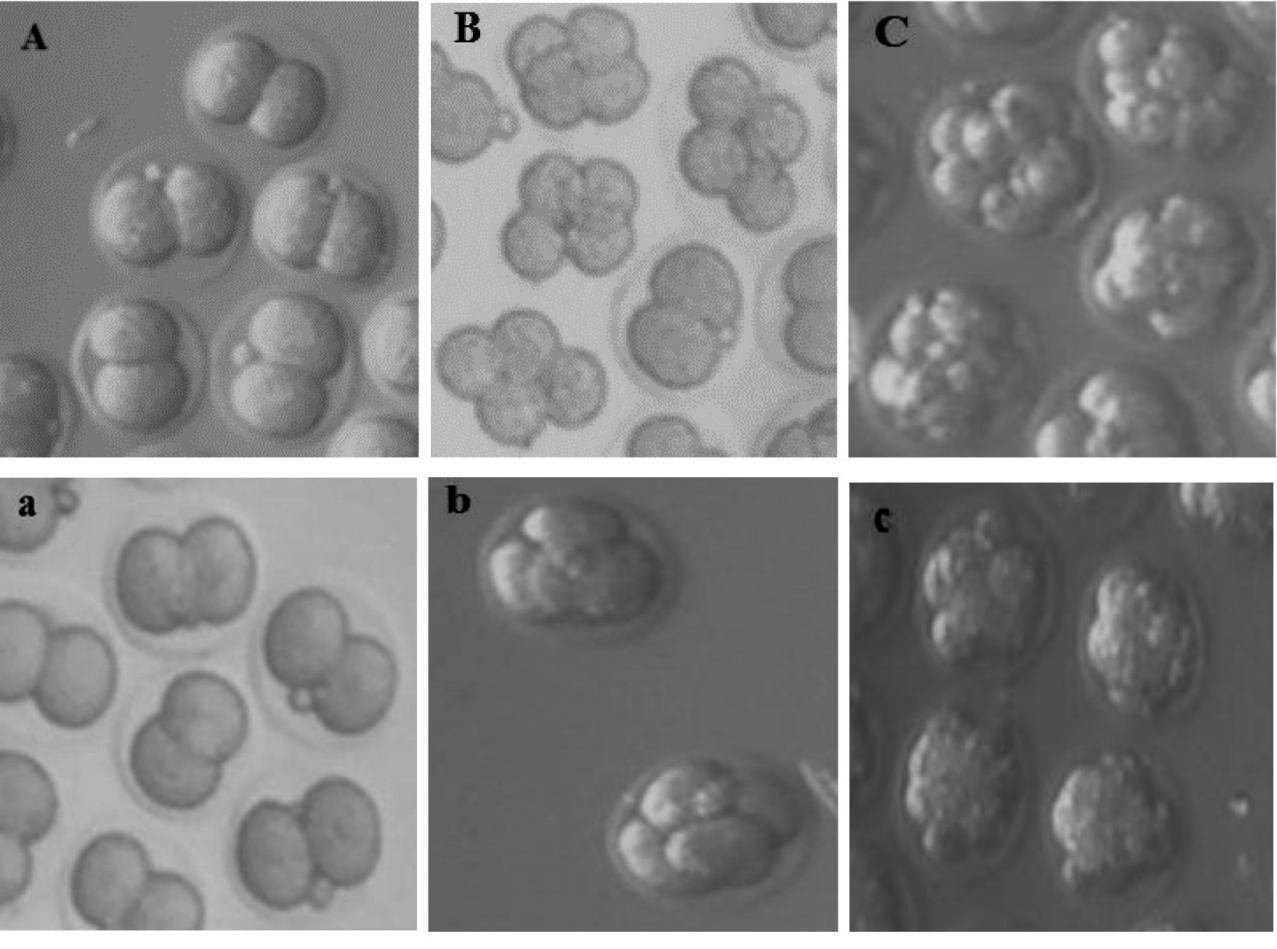
Different cleavage stages of fresh mouse embryos in the culture medium. A. 2-cell embryos, B. 4-cell embryos, C. 8-cell embryos. Different cleavage stages of vitrified mouse embryo 2 hours after thawing in the culture medium. a. 2-cell embryos, b. 4-cell embryos, and c. 8-cell embryos (magnification: ×200).

**Fig.2:**
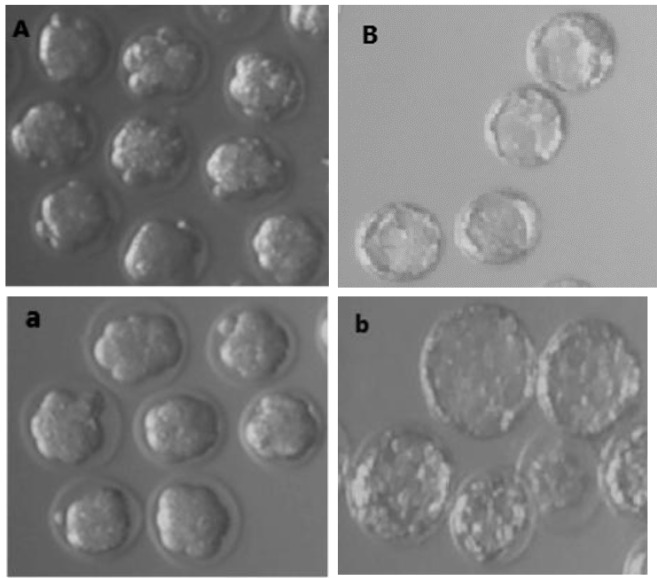
Different cleavage stages of fresh mouse embryos in the culture medium. A. Morula embryos, B. Blastocyst embryos. Different cleavage stages of vitrified mouse embryos 2 hours after thawing in the culture medium. a. Morula embryos and b. Blastocyst embryos (magnification: ×200).

Embryonic development, after vitrification and the subsequent thawing are summarized in Table 1. No statistically difference was observed in the post-vitrification survival rates of vitrified mouse embryos at the 2-cell (82.1%), 4-cell (83.2%), 8-cell (85.8%), morula (77%) and blastocyst (60.5%) stages (P>0.05) (Fig .1,2). The blastocyst formation and hatching rate for the vitrified 2-cell embryos group were the lowest, 56.5% ± 23.4 and 21.2% ± 14.7 respectively (P<0.05). On the other hand, hatching rate of blastocysts were highest (59.8% ± 25.7, P<0.05). Hatching rate of vitrified 4-cell, 8-cell and blastocysts were significantly higher than that of 2-cell embryos (P<0.05). 

**Table 1:** Mouse embryonic development after early cleavage-stage embryo vitrification-warming


Stage of vitrification	Embryos (n)	Survival rate (%) ± SD	Blastocysts formation (%) ± SD	Hatched(%) ± SD

Vitrified 2-cell	157	82.1%±16.8	56.5%±23.4	21.2%±14.7
Vitrified 4-cell	145	83.2%±11	79.8%±13.6	49.1%±25.5^*^
Vitrified 8-cell	166	85.8%±22.4	84%±21.2^*^	50.6%±25.7^*^
Vitrified morula	180	77%±17.8	75.8%±21	30%±16.3
Vitrified blastocyst	140	60.5%±22.3	-	59.8%±25.7^*^


* ; Significantly different from those of 2-cell embryos within the same column (P>0.05).

## Discussion

Despite the fact that there are many advances in the field of cryopreservation of embryo, there is no agreement on the optimal developmental stage for cryopreservation of embryo. In the current study, we vitrified different stages of mouse embryos through the Cryotop method. There was no significant difference in survival rate after thawing the vitrified embryos at 2-cell, 4-cell, 8-cell, morula and blastocyst stages. This finding is consistent with the results of studies performed by Zhou et al. (1) and Zhang et al. (4), which showed that there was no difference in survival rates among preimplantation mouse embryos. In contrast, Yan et al. (8) reported that mouse 2-cell embryos had the best tolerance for cryopreservation, but they used the open-pulled straw (OPS) method for vitrification. Differences in the survival rate may be due to the different strains of mice used in various studies. Investigators have shown that genotype could significantly influence post-thaw viability of frozen mouse embryos. In addition to genotype, such other factors as thawing temperature and the culture medium may also contribute to differences in the survival rates (17). 

Zhou et al. (1) demonstrated that early blastocyst stage is the most feasible stage for mouse embryo cryopreservation. Blastocyst is more complex and different from the earlier stages of development, whereby it contains an inner cell mass, a trophoblastic layer and a blastocoelic cavity. Regarding to various cell types presented in blastocyst, there is a greater chance for different metabolic activities and permeability when compared to the earlier stages of development (18). Generally, developmental stage of blastocyst (19), types of the cryoprotectants (20), exposure time to equilibration with vitrification solutions (19) and type of the carriers (21) can all affect the survival rates of the blastocyst. 

Although the survival rates for various embryonic stages were similar, in our study blastocyst formation in 2-cell stage embryos were significantly lower than that of 8-cell stage embryos. However, the rates of blastocyst formation for 4-cell, 8-cell and morula stages were similar. Zhang et al. (4), reported similar results to the demonstrated findings by Zhou et al. (1), conducted by OPS method, indicating that blastocyst rate of the vitrified 1-cell and 2-cell embryos were lower than those of the vitrified 4-cell, 8-cell and morula embryos. 

Graves-Herring and Boone (22) cryopreserved mouse 2-cell and 8-cell embryos in either an openor closed-Stripper Tip or an openor closed-CBS nozzle. The 8-cell provided a higher blastocyst rate than the 2-cell stage embryos, vitrified using the same manner. 

According to our research, hatching rate of 2-cell embryos was significantly lower than that of the 4-cell, 8-cell and blastocyst stages embryos. This finding correlates with that of Zhang et al. (4), indicating that hatching rate of the vitrified 2-cell embryos were significantly lower than that of the vitrified 8-cell embryos. In the vitrified 4-cell embryo group, hatching rate was significantly lower than that of the vitrified 8-cell embryo groups. Our study, however, showed a similar hatching rate for 4and 8-cell embryos. 

On the other hand, in a study performed by Păcală et al. (18), hatching rates of the morula stage embryos were higher than that of the blastocyst stage embryos. In this study, glycerol was used as a permeating cryoprotective agent. There is experimental proof that permeability co-efficiency of glycerol is increased from the 1-cell to the blastocyst stage in mice. After thawing, increase in glycerol absorption needs much time to efflux from the embryo (23). 

It must be noted that is essential for 2-cell embryos to be kept in culture medium for several days until they reach their blastocyst and hatching stages. 2-cell embryos also need to withstand with any kind of stress, induced by cold, being out of the incubator for investigations and potential recordings, and above all, the harmful effects of culture medium during this period. It is logic that some embryos sustained damage by various prevailing peripheral stresses, leading to lower overall blastocyst formation and decreased hatching rate. 

A study by Lane et al. (24) demonstrated that vitrification of hamster 2-cell embryos inhibited the activity of two transport proteins (Na+/H+ antiporter an HCO-/Clexchanger), which are responsible for the regulation of intracellular pH, playing a key regulatory role in metabolism, energy production, and cell division. It is proposed that similar reactions may affect mouse 2-cell embryos. 

## Conclusion

From the obtained results, it appears that developmental potential of mouse 2-cell embryos is more impacted by vitrification compared to 4-cell or 8-cell stage. The success of vitrification technique depends on the developmental stage of embryo and the utilized method. Cryotop method is suitable for vitrification of mouse cleavage-stage embryos from 2-cell stage to blastocyst, with the same effect on the survival rate. Embryos at 4-cell and 8-cell stages are at the most suitable options for vitrification, using cryotop method. They can show highest tolerance to the stress of freezing as well as thawing. They can also yield highest level of post-vitrification developmental competence among early cleavage stage embryos. 
